# Population genetic structure and post-LGM expansion of the plant bug *Nesidiocoris tenuis* (Hemiptera: Miridae) in China

**DOI:** 10.1038/srep26755

**Published:** 2016-05-27

**Authors:** Huaizhu Xun, Hu Li, Shujuan Li, Shujun Wei, Lijuan Zhang, Fan Song, Pei Jiang, Hailin Yang, Fei Han, Wanzhi Cai

**Affiliations:** 1 Department of Entomology, China Agricultural University, Beijing 100193, China; 2 Maricopa Agricultural Center, University of Arizona, Maricopa, AZ 85138, USA; 3 Institute of Plant and Environmental Protection, Beijing Academy of Agriculture and Forestry Sciences, Beijing 100097, China; 4 Cotton Research Institute, Chinese Academy of Agricultural Sciences/State Key Laboratory of Cotton Biology, Anyang, Henan 455000, China; 5 Yuxi subsidiary of Yunnan Tobacco Company, Yuxi, Yunnan 653100, China; 6 Department of Science and Technology, State Tobacco Monopoly Bureau, Beijing 100045, China

## Abstract

The plant bug, *Nesidiocoris tenuis* (Hemiptera: Miridae), is one of the most thermophilous dicyphines in agroecosystems and is widely distributed in China. Little is known regarding the genetic structure of *N. tenuis* and the effect of historical climatic fluctuations on *N. tenuis* populations. We analyzed partial sequences of three mitochondrial protein-coding genes (*COI, ND2* and *CytB*) and nuclear genes (*5.8S*, ITS2 and *28S*) for 516 specimens collected from 37 localities across China. Analyses of the combined mitochondrial dataset indicated that the Southwestern China group (SWC) was significantly differentiated from the remaining populations, other Chinese group (OC). Asymmetric migration and high level of gene flow across a long distance within the OC group was detected. The long-distance dispersal of *N. tenuis* might be affected by air currents and human interference. Both the neutrality tests and mismatch distributions revealed the occurrence of historical population expansion. Bayesian skyline plot analyses with two different substitution rates indicated that *N. tenuis* might follow the post-LGM (the Last Glacial Maximum) expansion pattern for temperate species. Pleistocene climatic fluctuation, complicated topography and anthropogenic factors, along with other ecological factors (e.g. temperature and air current) might have accounted for the current population structure of *N. tenuis.*

The plant bug *Nesidiocoris tenuis* is considered as a major natural enemy and has shown its beneficial role in control of several pests such as whiteflies, leafminers, aphids, thrips and spider mites^1^. This species also may feed on certain host plants (e.g. tobacco) causing flower abortion and viral diseases under prey shortage conditions^1^,^2^, but the relevant studies demonstrated that when the *N. tenuis* populations are well established, pest infestation is less severe^1^ and financial gain due to reduction in pest numbers greatly exceeds any losses^3^. *N. tenuis* is currently widely commercialized and mass supplied in southern and warm production areas^1^,^3^. Although the bug is small in size and exhibits limited ability for active dispersal^4^, it is widely distributed in China^5^. As one of the most thermophilous species of all dicyphines^6^, the range of *N. tenuis* covers various climate types, from temperate to subtropical and tropical region^4^. Thus, China provides excellent opportunity to study population genetics and demographic history of *N. tenuis*, due to, wide range of climate types and the presence of several refugia.

*N. tenuis* is a model organism of biological control potential, and knowledge of its population genetics and associated factors is fundamentally crucial for species management and conservation strategies. Multiple factors can influence population dynamics, genetic diversity and population structure^7^. For example, strong dispersal ability and human interference promote frequent gene flow between populations, which can decrease genetic subdivision within populations. In contrast, low gene flow between populations can lead to genetic subdivision of populations^8^, and genetic divergence arises from geographic barriers, adaptation to pesticides and other environmental factors^7^,^9^. The Quaternary Period has played an important role in shaping current distribution and genetic diversity of organisms on earth^10^. Sea level, ocean currents and peninsular climate have changed repeatedly during the Pleistocene, and organisms have shown diverse contraction and expansion patterns, e.g. some species went extinct, some recolonized in new locations, some survived in refugia and expanded after glacial periods^10^. To date, a number of studies have focused on the morphology, biological characteristics, thermal biology of *N. tenuis*, and its efficacy against pests^3^,^4^,^6^,^11^. However, the factors for population structure of *N. tenuis* in China remained unknown.

Herein we investigated its genetic diversity, population structure and demographic history using sequences of mitochondrial and nuclear data. The objectives of this study were to (1) reveal the genetic distribution of *N. tenuis* related to current factors (geographical barriers, ecological factors and human interference); and (2) investigate demographic history of *N. tenuis* affected by Pleistocene climate fluctuation in China.

## Results

### Population genetic diversity and structure

For the mitochondrial genes, 27 haplotypes for *COI*, 65 haplotypes for *ND2*, and 30 haplotypes for *CytB* were identified, respectively. A total of 130 haplotypes were detected in a combined mitochondrial dataset, which included 2, 226 bp of protein-coding regions (*COI*: 726 bp, *ND2*: 837 bp and *CytB*: 663 bp). Among these identified haplotypes, 102 were unique haplotypes and six haplotypes (H1, H4, H6, H12, H13 and H17) were most widely shared. The H1 haplotype was shared by 120 individuals and was detected in most populations. The H4, H6, H12, H13, H17 haplotypes accounted for 9.50%, 7.75%, 10.47%, 5.04%, and 6.40%, respectively. In addition, we observed 145 polymorphic sites, which were composed of 54 parsimony-informative sites (2.29%) and 91 singleton-variable sites (4.09%). High haplotype diversity (Hd) and low nucleotide diversity (Pi) were shown in all 37 populations (Table S1). The haplotype diversity ranged from 0.705 (TX) to 1.000 (XICH) with the average of 0.913, while the nucleotide diversity ranged from 0.0012 (TX) to 0.0052 (KM) with the average of 0.0030 (Table S1).

For the nuclear data, 509 sequences were successfully obtained with the length of 753 bp (*5.8S*: 82 bp, ITS2: 423 bp and *28S*: 248 bp). Compared to the results of the combined mitochondrial dataset, fewer haplotypes (HN = 30) and a lower level of genetic diversity (Hd = 0.183) were observed in the nuclear data. In addition, we observed 34 polymorphic sites, which were composed of one parsimony-informative site (0.13%) and 33 singleton-variable sites (4.38%).

**Figure 1:**
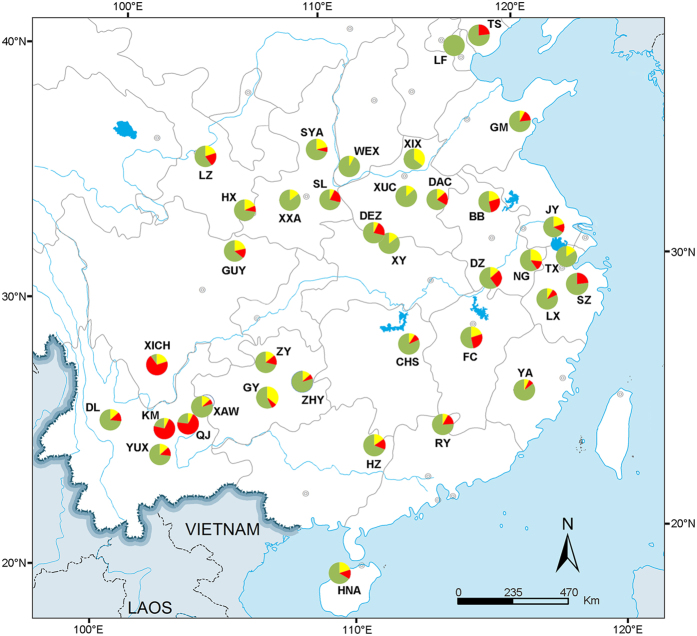
Results of spatial clustering of the *Nesidiocoris tenuis* individuals analysis in the programs BAPS based on the combined mitochondrial dataset. Pie chart with color indicates the proportion of three predicated clusters in each population. Upper-case letters are the abbreviation of 37 sampled locations. KM: Kunming, Yunnan; QJ: Qujing, Yunnan; XICH: Xichang, Sichuan; HNA: Danzhou, Hainan; YUX: Yuxi, Yunnan; DL: Dali, Yunnan; XAW: Xuanwei, Yunnan; YA: Yong’an, Fujian; HZ: Hezhou, Guangxi; XIX: Xinxiang, Henan; GY: Guiyang, Guizhou; ZY: Zunyi, Guizhou; ZHY: Zhenyuan, Guizhou; GM: Gaomi, Shandong; TS: Tangshan, Hebei; WEX: Wenxi, Shanxi; RY: Ruyuan, Guangdong; TX: Tongxiang, Zhejiang; SZ: Shengzhou, Zhejiang; JY: Jiangyin, Jiangsu; LX: Lanxi, Zhejiang; DZ: Dongzhi, Anhui; CHS: Changsha, Hunan; FC: Fengcheng, Jiangxi; BB: Bengbu, Anhui; NG: Ningguo, Anhui; XUC: Xuchang, Henan; DAC: Dancheng, Henan; DEZ: Dengzhou, Henan; XY: Xiangyang, Hubei; SL: Shangluo, Shaanxi; SYA: Yan’an, Shaanxi; XXA: Xi’an, Shaanxi; GUY: Guangyuan, Sichuan; HX: Huixian, Gansu; LZ: Lanzhou, Gansu; LF: Langfang, Hebei. Map was generated with ArcGIS 10.0 (http://www.esri.com/software/arcgis/arcgis-for-desktop) and modified with Adobe Photoshop CS6 (http://www.adobe.com/products/photoshop).

For the combined mitochondrial dataset, the spatially explicit BAPS model for clustering of individuals identified three clusters in the 37 populations (Fig. 1). The first cluster (red color in Fig. 1), mainly distributed in three populations from Southwestern China, and accounted for 71.43% (KM), 70.00% (XICH), and 69.23% (QJ) of samples at each location. The second cluster (green color in Fig. 1) was distributed in the rest of the 34 populations as a majority (more than 53.33%), and the third cluster (yellow color in Fig. 1) was distributed as a minority (less than 35.7%).

The pairwise *F*_*ST*_ values for genetic differentiation varied from −0.076 to 0.254 based on the combined mitochondrial dataset, and from −0.090 to 0.170 based on the nuclear data. For the combined mitochondrial dataset, three populations (KM, QJ and XICH) exhibited the most significant genetic differentiation (Table S2). Moreover, the pairwise *F*_*ST*_ values among KM, QJ and XICH population were relatively low and non-significant, indicating that high gene flow existed among these populations.

**Figure 2:**
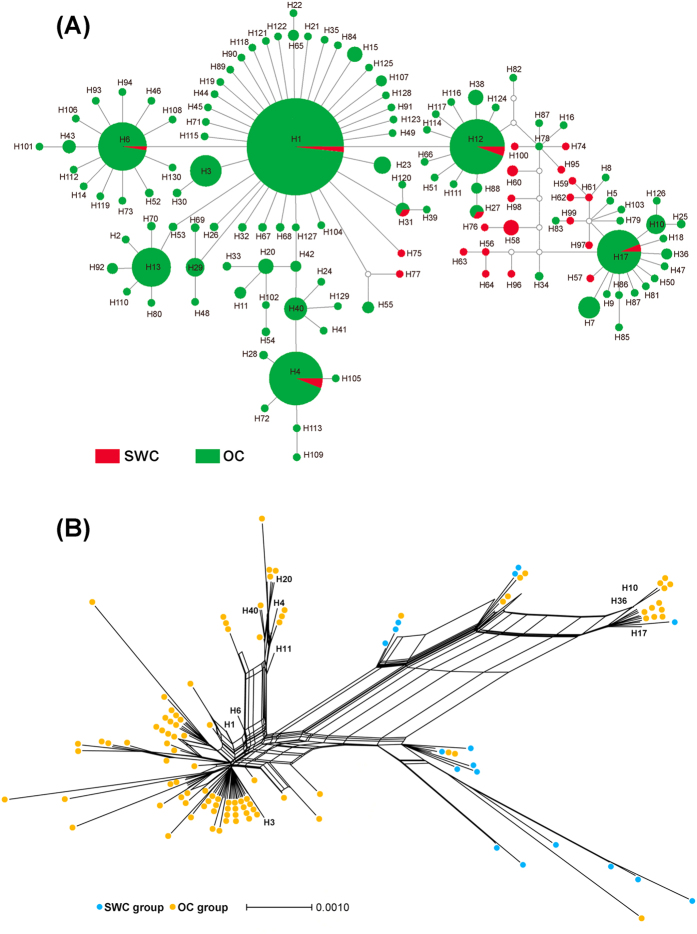
The median-joining network and splits network for *Nesidiocoris tenuis* based on the combined mitochondrial dataset. (**A**) Haplotype network with the median-joining algorithm. The circle size of haplotype denotes the number of observed individuals. White dots represent lost haplotypes. The shortest trees with median vectors were shown. (**B**) The splits network constructed by the neighbor-net method. The “H” with a number represents the haplotype shared by different populations.

The median-joining network construction among the mitochondrial haplotypes also identified two groups (Fig. 2A), which was consistent with the result of BAPS analysis (Fig. 1). One group was the clustering of three Southwestern populations (KM, QJ and XICH) (red in Fig. 2A), which contained large amount of unique haplotypes and a few shared haplotypes. The other group included six most frequent haplotypes and their derived haplotypes from remaining populations (green in Fig. 2A). The ancestral haplotype H1 is represented by the largest circle. For the nuclear data, the median-joining network construction showed a big star-like shape with the H1 haplotype as the center, along with its derived haplotypes and no obvious geographically separated clusters (Fig. S2). Furthermore, the analysis using SplitsTree for the combined mitochondrial dataset revealed that a minor group was separated from the other haplotypes (blue color in Fig. 2B). This minor group was composed of haplotypes from three Southwestern populations (KM, QJ and XICH), corresponding to the result in Bayesian tree (Fig. S4) based on the combined mitochondrial haplotypes.

The results above suggested two well-supported groups as follows: the SWC group included three populations from Southwestern China (KM, QJ and XICH); and the OC group included the remaining 34 populations in China (JY, LX, FC, CHS, DZ, YUX, XAW, DL, YA, HZ, XIX, GY, ZY, ZHY, GM, TS, WEX, HNA, RY, TX, SZ, BB, NG, XUC, DAC, DEZ, XY, SL, SYA, XXA, GUY, HX, LZ, LF).

**Figure 3:**
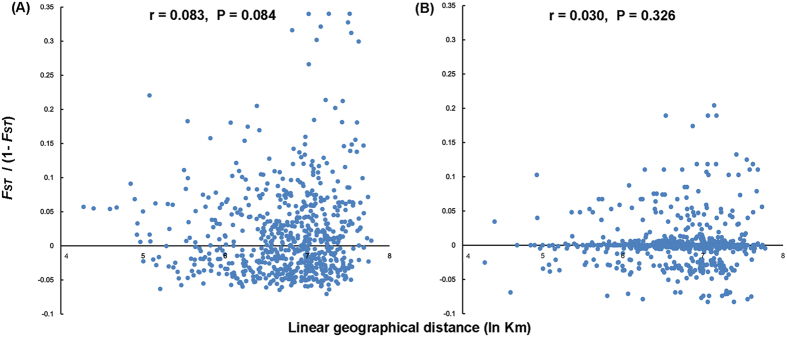
Scatter plots of genetic distance vs. geographic distance for pairwise population comparisons based on the combined mitochondrial dataset (**A**) and the nuclear data (**B**). Both analyses are calculated from 1,000 randomizations.

Non-significant isolation-by-distance (IBD) effect was detected among populations of *N. tenuis*, based on analyses of Mantel tests for both the combined mitochondrial dataset (r = 0.083, P = 0.084; Fig. 3A) and nuclear data (r = 0.030, P = 0.326; Fig. 3B).

### Hierarchical analysis of molecular variance and test to group definition

**Figure 4:**
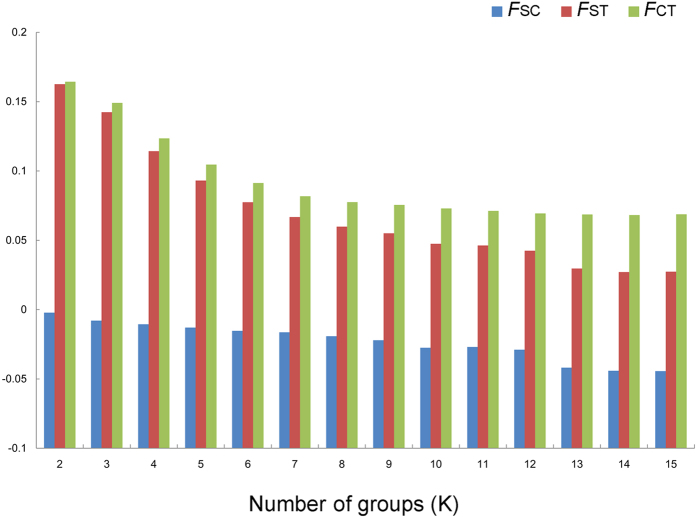
Fixation indices correspond to the number of groups (K) defined by SAMOVA analysis based on the combined mitochondrial dataset.

For the combined mitochondrial dataset, significant genetic structure among all populations (Φ_ST_ = 0.01809, P < 0.05) was observed. AMOVA analysis showed that 1.81% of the variation was partitioned among populations and the other 98.19% was within populations. SAMOVA analysis revealed that the *F*_*CT*_ value reached the peak at K = 2 and decreased subsequently (Fig. 4), which supported a two-group structure. The pairwise *F*_*ST*_ value (*F*_*ST*_ = 0.1295, P < 0.001) also showed the significant genetic differentiation between two defined groups (SWC and OC). Moreover, the three-level AMOVA analysis indicated extremely significant structure between two groups (Φ_CT_ = 0.1298, P < 0.001), with 12.97% genetic variation.

For the nuclear data, SAMOVA analysis demonstrated that the *F*_*CT*_ was highest at eight groups when K value increased from 2 to 15 (Fig. S3). Non-significant genetic structure between two groups and most genetic variation (99.72%) within populations were detected using AMOVA analysis (Table 1).

Analyses on divergence of *COI* sequence indicate that genetic distances between the populations are in the range from 0.06% to 0.38%, and genetic distance between established groups SWC v OC is 0.28%.

### Gene flow

The median-joining network analysis demonstrated that some haplotypes were widely shared, indicating the occurrence of frequent gene flow across China despite of long geographic distance (Fig. 2A and Fig. S2). It appeared that SWC group was significantly differentiated from OC group, and high level of gene flow existed between populations in OC group (Table S2). Therefore, analysis for direction of gene flow was performed within OC group.

**Figure 5:**
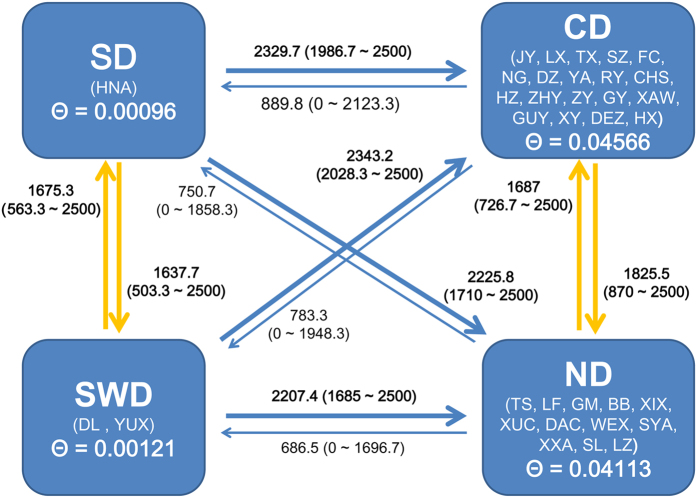
Estimates of the migration (*M* and *θ*) among four geographic districts of *Nesidiocoris tenuis* based on the combined mitochondrial dataset. *θ* represents the mutation-scaled population size, and *M* indicates the mutation-scaled migration rate.

When four geographical districts were analyzed, estimates of effective population size (*θ*) were consistently low and ranged from 0.00096 for Southern District (SD) to 0.04566 for Central District (CD) (Fig. 5). Estimates of migration rate between regions were bi-directional and relatively high, which ranged from 686.5 to 2343.2. The highest *M* value (2343.2) was found for samples from Southwestern District (SWD) to CD, while the lowest *M* value (686.5) was for samples from the Northern District (ND) to SWD. Significant asymmetrical migration rates among long-distance district pairs, e.g. SWD-CD pair, were discovered by non-overlapping 95% confidence intervals of each estimate.

When the *θ* values and *M* values were translated into effective migrants per generation (*Nem* = *θM*), most migrants into CD (290.39) and ND (257.42) were obtained (Table S3). Estimate of migrants leaving out of SD and SWD was considerably high with the values of 199.90 and 199.39, respectively; however, extremely low values were detected for migrants entering into SD (3.18) and SWD (3.76). Therefore, SD and SWD were regarded as the major producers of migrants.

### Demographic history

When two groups were defined, Fu and Li’s D*, Fu and Li’s F* and Tajima’s D values were not significantly negative in the SWC group, but were significantly negative in the OC group. When all samples were considered as one group, three neutrality tests were statistically significant (P < 0.02), which indicated that *N. tenuis* experienced recent demographic expansion (Table 2).

**Figure 6:**
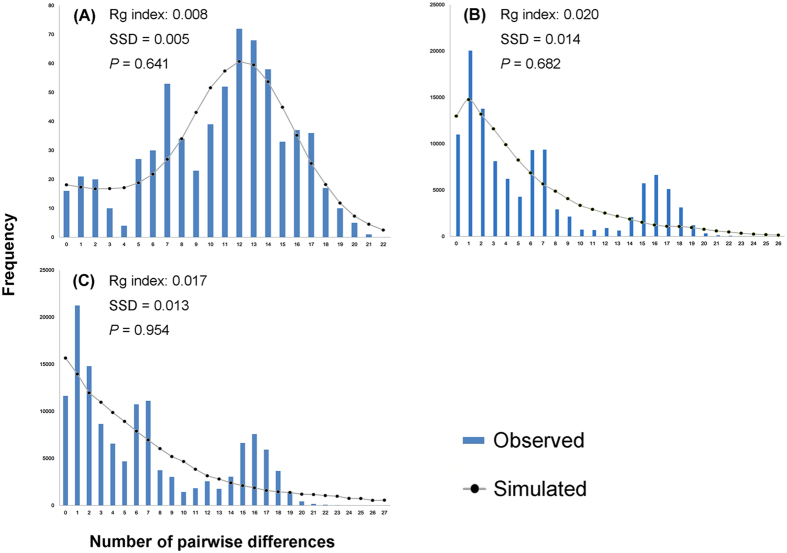
Mismatch distributions of the combined mitochondrial dataset in the SWC group (**A**), OC group (**B**) and all samples of *Nesidiocoris tenuis* from China (**C**). X-axis represents the number of pairwise differences, and Y-axis represents the relative frequencies of pairwise comparisons.

For SWC group, the multimodal mismatch distribution demonstrated that the group was under a model of population stability, which was consistent with the results of neutrality tests (Fig. 6A). OC group and whole populations were characterized by left skewed unimodal mismatch distributions, suggesting the population expansion models. Small *Rg* values but non-significant SSD values also indicated sudden population expansion, which was in line with the results of neutrality tests (Fig. 6B,C).

**Figure 7:**
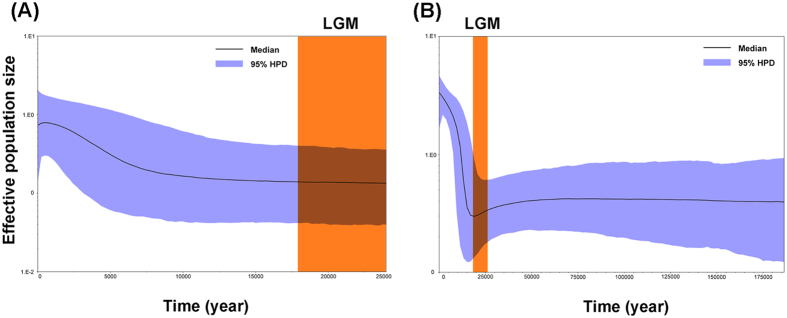
Demographic history of *Nesidiocoris tenuis* reconstructed using Bayesian skyline plots based on *COI* gene with substitution rate of 0.0177 (**A**) and the combined mitochondrial dataset with substitution rate of 0.0115 (**B**). X-axis is the timescale before present, and Y-axis is the estimated effective population size. Solid curves indicate median effective population size; the shaded range indicates 95% highest posterior density (HPD) intervals. LGM represents Last Glacial Maximum.

When using the Bayesian skyline plot with the substitution rate at 1.77% per million years based on *COI* gene, the effective population size of whole populations remained stable for a long period, and was followed by expansion about 9,000 years ago in a slow manner (Fig. 7A). Analysis with a slower substitution rate at 1.15% per million years based on the combined mitochondrial dataset also supported a growth pattern of effective population size through time, in which a sharp rise occurred about 15,000 years ago and later expanded in a considerably fast manner (Fig. 7B). Before the sharp rise, the population size remained stable over a long period, but a small decrease started after the last inter-glacial (LIG) and throughout the last glacial maximum^12^ (LGM: 26 Kya ~ 18 Kya; Kya: 1000 years ago). Both analyses suggested the population expansion after the LGM, which was the coldest period dating back.

## Discussion

According to Hebert^13^, more than 98% of species pairs exceed 2% sequence divergence, but divergences of *COI* sequence in our study are much less than 2%, suggesting that our result is in the context of divergences within species. Our analyses based on the combined mitochondrial dataset suggested that SWC group was significantly differentiated from OC group, while the analyses of nuclear data showed an extensive level of shared haplotypes between groups. Incongruence between mitochondrial and nuclear data is a common issue for phylogeographic study, which might be caused by various factors, e.g. potentially sex-biased dispersal, incomplete lineage sorting or recent secondary contact^14^,^15^,^16^,^17^. If males exhibited higher ability to disperse due to the natal philoparty of females, the expected result would be significant IBD effect based on the mitochondrial dataset and non-significant IBD effect based on nuclear data^17^,^18^. However, our analyses for both the mitochondrial and nuclear data displayed non-significant IDB effects, indicating that the incongruence could not be mainly explained by sex-biased dispersal (e.g. the higher male dispersal ability). In addition, we found that common haplotypes were widely shared by populations, and descendant haplotypes coexisted with ancestral haplotypes. This genetic pattern could be affected either by secondary contact or incomplete lineage sorting^19^. If the observed mitochondrial DNA pattern resulted from incomplete lineage sorting, a shallower divergence pattern would be expected in the nuclear data, given the larger effective population size and thus a longer sorting time^20^. However, no distinct genetic differentiation was revealed in nuclear data (Fig. S2), suggesting that incomplete lineage sorting was unlikely to be the primary factor.

*Nesidiocoris tenuis* was the most thermophilous species of all dicyphines in the Mediterranean region^6^, and our Bayesian skyline plot results suggested that this species might rapidly expand from refugial areas and establish secondary contact during the warm period after the LGM. Thus, the recent secondary contact followed by gene flow was mainly responsible for the incongruence between mitochondrial and nuclear data, which was reported in the vinous-throated parrotbill *Paradoxornis webbianus*^21^. Another apparent indication of recent secondary contact was found in the Hainan population (HNA). HNA population was expected to separate from the populations in the mainland due to its isolation by the Qiongzhou strait. However, our analysis revealed non-significant *F*_*ST*_ value between HNA and most continental populations. The most common haplotype (H1) was detected in the HNA population as well (Fig. 1). Such case was probably attributed to secondary contact by historically isolated refugia because the Quaternary glacial/interglacial cycles could cause the connection and isolation between Hainan Island and mainland repeatedly^22^.

The SWC group was located in the Hengduan Mountains, which were considered as one biodiversity hot-spot because of their temperature and humidity, specific topography, and refugia for many organisms^23^,^24^, especially in the suitable habitats for SWC group (e.g. higher genetic diversity in Table 2). Many unique haplotypes in the SWC group were not observed in other locations in the OC group, indicating habitat quality variation and geographic barriers might largely contribute to genetic division between SWC group and OC group. In addition, small local extinctions and different selection pressure might arise the phylogeographic breaks without geographic barriers^25^, e.g. SWC group and other sites located in the Hengduan Mountains (XAW, YUX and DL). The unique haplotypes in the SWC group were favored in local habitats because of selection pressure^25^, thus phylogeographic breaks could persist despite much gene flow.

*N. tenuis* populations in the OC group didn’t reveal a strong genetic division within a large range of locations, although its small size and limited ability for active dispersal might be expected to prevent gene flow. No correlation between genetic distance [*F*_*ST*_/(1 − *F*_*ST*_)] and geographic distance (ln Km) were determined using Mantel tests based on both molecular datasets, indicating IBD effect was non-significant among populations of *N. tenuis* in China (Fig. 3). Gene flow among populations across a long distance was observed within this group. The lowest pairwise *F*_*ST*_ values (−0.076) between CHS population and TS population (geographic distance = 1385.467 Km), and a non-significant *F*_*ST*_ value (0.007) was detected between the two furthest populations (TS and HNA; geographic distance = 2386.816 Km). We therefore speculated the homogeneity of populations was likely related to long-distance dispersal by human interference. It might be possible that biological companies introduced *N. tenuis* into different locations as a biological control agent and long-distance dispersal might be carried out due to the distribution chain operational in many countries^11^,^26^. Another possible human interference might be with respect to the frequent trade of crops, e.g. tobacco. Host plant dispersal could promote passive invading that was reported in the oriental fruit moth *Grapholita molesta*^27^. Likewise, a similar situation was reported for other small and sedentary species, e.g., booklouse *Liposcelis bostrychophila* was capable of long-distance dispersal (over 15,000 km) due to human-transport^28^, and the red flour beetle *Tribolium castaneum* widely colonized grain storages primarily through anthropogenic dispersal^29^.

Furthermore, our gene flow analyses confirmed long-distance dispersal, which demonstrated asymmetrical migration and high migration rates (Fig. 5). High migration rate was an indication of historically recurring gene flow, resulting from ancestral haplotypes persisting throughout glacial/interglacial periods and high frequencies of common haplotypes among regions^30^. A great number of migrants were detected moving from southern and southwestern districts to the central and northern districts of China, while rare migrants were obtained in the reverse direction (Table S3). The ‘South to North’ dispersal routes might be affected by thermal activity and air currents. Hughes *et al*. had shown limited dispersal abilities of *N. tenuis* from autumn to spring in certain temperate regions, while some dispersal would be possible in the summer^4^. Many small-sized insects could use air currents to carry out long-distance dispersal^31^. Our dispersal pattern of *N. tenuis* was consistent with that of the Asian subtropical monsoon, which occurs from southern and southwestern to northern direction in spring and summer^31^.

A variety of analyses indicated recent demographic expansion events for *N. tenuis*. Mitochondrial and nuclear network construction showed multiple star-like shapes, which implied that *N. tenuis* experienced population expansion events more than once^32^. Both the neutrality tests (Table 2) and mismatch distributions (Fig. 6C) indicated that *N. tenuis* experienced recent demographic expansion events. The results were in line with the estimates using Bayesian skyline plots with different rates (Fig. 7A,B). The estimated time of the expansion event, using the *COI* gene, was about 9 Kya (after the LGM). The interspecific substitution rate used in the previous *COI* analysis was much lower than mutation rate within species due to the delayed effects of purifying selection^33^. Consequently, our estimated population expansion time might be earlier. Based on the combined mitochondrial dataset, *N. tenuis* populations were estimated to start expanding nearly 15 Kya, which was consistent with the result of population expansion after the LGM. Although the results from the substitution rates based on *COI* gene and the combined mitochondrial dataset were different, the same post-LGM expansion pattern was obtained.

Unlike the expansion during the LIG to LGM transition, which has been observed in other organisms in Asia^34^,^35^, our results were consistent with the general consensus on a classic post-LGM expansion pattern for most thermophilous species in Europe. Moreover, our Bayesian skyline plot result based on the combined mitochondrial dataset demonstrated a contraction in population size during the LIG to LGM transition and throughout LGM period. We speculated that the demographic expansion of *N. tenuis* after the LGM was related to climate changes, because temperate species may present contracted distribution during glacial periods and experience range expansions during interglacial periods^10^. *N. tenuis* was proved to be the most thermophilous of all dicyphines in the Mediterranean region^6^ and lacks cold tolerance^3^,^4^. Thereby the numbers of beneficial habitats were increasing as the climate became warmer after the LGM. Other reasons for the demographic expansion after the LGM might be the wider distribution of the prey^36^ and human population expansions^37^ (~7 Kya), which provided food sources and long-distance dispersal to support rapid population expansion. The post-LGM expansion pattern was also observed in other insects such as the stable fly *Stomoxys calcitrans*^38^ and locusts *Locusta migratoria*^39^, whose effective population size rapidly increased approximately 12 Kya ~ 7 Kya and 5 Kya, respectively.

In summary, a combination of historical factors (the Quaternary glacials/interglacials cycles), ecological factors (specific topography and comfortable climate) and anthropogenic factors (passive dispersal ability by long-distance) might have shaped the current population structure pattern and dispersal routes of *N. Tenuis.*

## Materials and Methods

### Sample collection, DNA extraction and sequencing

In this study, a total of 516 adult *N. tenuis* individuals were collected from 37 locations, covering all representative distributions in China^5^. All specimens were stored in absolute ethanol at −20 °C until DNA extraction. Genomic DNA was extracted from single adult insect using a TIANamp genomic DNA kit (TIANGEN Biotech Co., Ltd., Beijing, China). The abdomen was removed prior to DNA extraction. Voucher specimens were deposited at the Entomological Museum of China Agricultural University, Beijing, China.

Considering the genetic variability of different genes, three mitochondrial protein-coding genes (a partial sequence of *COI*, a fragment of *ND2* and partial *CytB* gene) and nuclear genes (partial *5.8S*, complete ITS2 and partial *28S* region) were used as molecular markers. Primers for three mitochondrial genes were specifically designed based on the complete mitochondrial genome of *N. tenuis*^40^, while primer pairs of nuclear data were used from a previous study^41^ (Table S4). The PCR amplifications were performed using Takara rTaq polymerase (Takara Biomedical, Japan) in a total volume of 25μl with the following conditions: an initial denaturation at 94 °C for 50 s, followed by 35–40 cycles with 30 s at 94 °C, 30 s at 45–52 °C, and 1–2 min at 72 °C, and a final extension step at 72 °C for 5 min. PCR products were visualized on 1.0% agarose gels under UV light. Purified PCR products were sequenced in both directions by Ruibo Biotechnology Co., Ltd (Beijing, China). All sequences have been deposited in GenBank under accession numbers, KF017246 - KF017265 and KT598365 - KT598371 for *COI*, KT587084 - KT587148 for *ND2*, KT587054 - KT587083 for *CytB*, and KT587149 - KT587178 for ITS2 plus partial *5.8S* and *28S*.

### Population genetic diversity and structure

Sequences of mitochondrial and nuclear markers were aligned independently using Clustal W implemented in MEGA version 5 42 with default parameters. Alignment of nucleotide sequences of the mitochondrial protein-coding genes (*COI, ND2* and *CytB*) were inferred from the amino acid alignment. The number of polymorphic sites (S), the number of haplotypes (HN), haplotype diversity (Hd), nucleotide diversity (Pi) and average number of nucleotide differences (K) for each location were calculated using Arlequin version 3.5 43.

Several approaches were used in order to understand the population genetic structure of *N. tenuis*. The spatially explicit BAPS model for clustering of individuals, implemented in BAPS version 6.0^44^, was performed for mitochondrial genes. 20 runs (K = 20) were performed to ensure consistency and convergence of the results. Pairwise *F*_*ST*_ analysis with 10,000 permutations was also calculated using Arlequin version 3.5 43 to estimate the genetic differentiation for population pairs. The median-joining networks among haplotypes were further reconstructed using Network version 4.6.1.3 45, based on the combined mitochondrial dataset and nuclear data independently.

To reveal the relationships among mitochondrial haplotypes, the splits network was constructed using SplitsTree version 4.13.1 46. The neighbor-net method was used under the p-distance model. Additionally, a phylogenetic tree based on the combined mitochondrial dataset was constructed with Bayesian inference (BI) using MrBayes version 3.2.1 47. The plant bug, *Trigonotylus caelestialium*, was chosen as the out-group^48^. Separate partitions were created for each gene in the combined mitochondrial dataset with the best-fit model that was determined under the Akaike Information Criterion using jModelTest version 0.1.1 49. The best-fit model was GTR+I+G for *ND2*, GTR+G for *COI* and GTR+G for *CytB*, respectively. The analysis was performed with two runs and four chains for 15 million generations, and the chains were sampled every 1000 generations. The first 25% of samples were discarded as burn-in.

To detect the effect of geographical isolation, Mantel tests^50^ with 1,000 randomizations for both the combined mitochondrial dataset and nuclear data were performed. The correlations of matrix of genetic distances [*F*_*ST*_/(1 − *F*_*ST*_)] vs. linear geographic distance (ln Km) were investigated using Mantel tests implemented in the software Arlequin version 3.5^43^.

### Hierarchical analysis of molecular variance and test to group definition

The spatial analysis of molecular variance (SAMOVA) was performed using SAMOVA version 1.0 51. The number of groups ranged from 2 to 15, and the values of fixation indices were compared among different group numbers with 1,000 permutations. To test the rationality of defined groups, pairwise *F*_*ST*_ values between defined groups and hierarchical analyses of molecular variance (AMOVA) with 10,000 permutations were executed using Arlequin version 3.5^43^ for both the combined mitochondrial dataset and nuclear data. To confirm our study in the context of divergences within species, analyses on divergence of *COI* sequence (DNA barcoding marker) were performed using MEGA version 5^42^ with the Kimura-two-parameter (K2P) model.

### Gene flow

Four districts were defined based on the zoogeographic regions of China^52^,^53^ to infer asymmetric dispersal accurately and present gene flow intuitively. Definitions were as follows (population codes): (1) Southern District (SD): HNA; (2) Central District (CD): JY, LX, TX, SZ, FC, NG, DZ, YA, RY, CHS, HZ, ZHY, ZY, GY, XAW, GUY, XY, DEZ and HX; (3) Northern District (ND): TS, LF, GM, BB, XIX, XUC, DAC, WEX, SYA, XXA, SL and LZ; and (4) Southwestern District (SWD): DL and YUX.

To estimate effective number of migrants entering and leaving each region per generation (*θM*), the mutation-scaled population size (*θ*; *θ* = *Neμ*, where *Ne* = effective population size and *μ* = mutation rate per generation) and the mutation-scaled migration rate (*M*; *M* = *m*/*μ*, where *m* = migration rate) were estimated using Bayesian inference implemented in Migrate version 3.6.4^54^. The first run was estimated from *F*_*ST*_ values, and three subsequent runs were started with *θ* and *M* from the previous run to confirm the consistency of results. For each run, one long chain with five independent replicates was contained, other parameters were as follows: long-inc = 20, longsample = 1,000,000, burn-in = 100,000. To increase the efficiency of the MCMC, four heating chains were used with approximately exponential increasing temperatures at 1.0, 1.5, 3.0, and 1,000,000. The final estimates of parameters along with 95% confidence intervals were reported here.

### Demographic history

Multiple approaches were explored to investigate the demographic history of *N. tenuis*. Neutrality analyses of Fu and Li’s F*, Fu and Li’s D* and Tajima’s D for the defined groups and whole populations were calculated using DnaSP version 5.0 program^55^. Under the assumption of neutrality, the population expansion produced a significantly negative value, whereas processes such as a population subdivision or recent population bottleneck were reflected in significantly positive values. Another method, pairwise mismatch distributions using Arlequin version 3.5 43, was used to infer whether demographic expansions had occurred. Unimodal mismatch distributions represented expanding populations, while multimodal formats revealed populations with relatively constant-size^37^. In addition, the statistics of the raggedness (*rg*) index of the observed distribution and the sum of square deviations (SSD) between the observed and the expected mismatch were also calculated using Arlequin version 3.5 43. A small value of the *rg* index indicated that populations had undergone recent demographic expansions, and a significant value of SSD was an indication of population stability^38^.

To estimating population expansion through time, Bayesian skyline plots were implemented in BEAST version 1.6.1 56. For the *COI* gene, a substitution rate of 1.77% per million years^57^ and the GTR+G model were adopted. The Markov Chain length was set to 300 million generations under an uncorrelated lognormal relaxed clock model, which allowed rate variation among branches. Samples were taken every 10,000 steps. The piecewise-linear skyline model for Bayesian skyline coalescent tree priors was selected and otherwise default parameters were used. The result of Bayesian skyline plot was checked and analyzed using Tracer version 1.4 56 with a burn-in of 10%. For the combined mitochondrial dataset, a slower substitution rate with 1.15% per million years^58^ and the GTR+I+G model were adopted.

## Additional Information

**How to cite this article**: Xun, H. *et al*. Population genetic structure and post-LGM expansion of the plant bug *Nesidiocoris tenuis* (Hemiptera: Miridae) in China. *Sci. Rep.* **6**, 26755; doi: 10.1038/srep26755 (2016).

## Supplementary Material

Supplementary Information

## Figures and Tables

**Table 1:** Hierarchical analysis of molecular variance (AMOVA) for *Nesidiocoris tenuis* based on the combined mitochondrial dataset and the nuclear data.

Gene	Source of variation	d.f.	SS	Percentage	Fixation indx
*COI*+*ND2*+*Cytb*	Two level				
	Among populations	36	148.376	1.81	
	Within populations	479	1571.002	98.19	Φ_ST_ = 0.0181*
	Three levels				
	Among groups	1	36.723	12.97	Φ_CT_ = 0.1298***
	Among populations within groups	35	111.653	-0.17	Φ_SC_ = −0.0020
	Within populations	479	1571.002	87.20	Φ_ST_ = 0.1280*
*5.8S*+ITS2+*28S*	Two level				
	Among populations	36	4.075	0.28	
	Within populations	472	51.442	99.72	Φ_ST_ = 0.0028
	Three levels				
	Among groups	1	0.178	0.77	Φ_CT_ = 0.0077
	Among populations within groups	35	3.896	0.15	Φ_SC_ = 0.0016
	Within populations	472	51.442	99.08	Φ_ST_ = 0.0092

^*^P < 0.05; ^**^P < 0.02; ^***^P < 0.001. d.f., degree of freedom; SS, sum of squares.

**Table 2:** Genetic diversity and demographic analysis for two defined groups (SWC and OC groups) and all samples of *Nesidiocoris tenuis* based on the combined mitochondrial dataset.

Parameter	SWC group	OC group	All samples
Hd	0.976	0.904	0.912
Pi	0.0048	0.0028	0.0030
Tajima’s D	−0.112	−1.998*	−2.057*
Fu and Li’s D*	−0.277	−11.268**	−10.598**
Fu and Li’s F*	−0.261	−7.703**	−7.240**

^*^P < 0.05; **P < 0.02. Hd, haplotype diversity; Pi, nucleotide diversity.
