# Supplementary material for: Population genetic structure and post-LGM expansion of the plant bug Nesidiocoris tenuis (Hemiptera: Miridae) in China
Source: Sci Rep. 2016 May 27;6:26755. doi: 10.1038/srep26755 (PMC4882614; doi:10.1038/srep26755)

**Population genetic structure and post-LGM expansion of the plant bug *Nesidiocoris tenuis* in China**

Huaizhu Xun^1^, Hu Li^1§^, Shujuan Li^2^, Shujun Wei^3^, Lijuan Zhang^4^, Fan Song^1^, Pei Jiang^1^, Hailin Yang^5^, Fei Han^6^ and Wanzhi Cai^1§^

^1^Department of Entomology, China Agricultural University, Beijing 100193, China

^2^Maricopa Agricultural Center, University of Arizona, Maricopa, AZ 85138, USA

^3^Institute of Plant and Environmental Protection, Beijing Academy of Agriculture and Forestry Sciences, Beijing 100097, China

^4^Cotton Research Institute, Chinese Academy of Agricultural Sciences /State Key Laboratory of Cotton Biology, Anyang, Henan 455000, China

^5^Yuxi subsidiary of Yunnan Tobacco Company, Yuxi, Yunnan 653100, China

^6^Department of Science and Technology, State Tobacco Monopoly Bureau, Beijing 100045, China

^§^Correspondence and requests for materials should be addressed to W. C. ([caiwz@cau.edu.cn](mailto:caiwz@cau.edu.cn)) and H. L. (tigerleecau@hotmail.com).

**Supplementary information**

**Supplementary Table S1.** **Geographical details and general genetic features of 37 populations of *Nesidiocoris tenuis*.**

| **Group** | **Code** | **Sampling location** | **Collection date** | **Longitude**  **(E)** | **Latitude**  **(N)** | **Elevation (m)** | **Host** | **N** | **HN** | **Hd** | **Pi** |
| --- | --- | --- | --- | --- | --- | --- | --- | --- | --- | --- | --- |
| **SWC** | KM | Kunming, Yunnan Province | 2012/6/21 | 102.7503° | 25.2946° | 2097.73 | Tobacco | 14 | 12 | 0.978±0.035 | 0.0052±0.0028 |
|  | QJ | Qujing, Yunnan Province | 2012/6/25 | 103.6925° | 25.5651° | 1931.17 | Tobacco | 13 | 10 | 0.949±0.050 | 0.0041±0.0023 |
|  | XICH | Xichang, Sichuan Province | 2013/9/7 | 102.2203° | 27.7196° | 1506.00 | Tobacco | 10 | 10 | 1.000±0.045 | 0.0050±0.0028 |
| **OC** | HNA | Danzhou, Hainan Province | 2013/6/2 | 109.4791° | 19.7996° | 29.00 | Tobacco | 15 | 8 | 0.914±0.043 | 0.0031±0.0017 |
|  | YUX | Yuxi, Yunnan Province | 2012/6/23 | 102.5080° | 24.3507° | 1643.00 | Tobacco | 15 | 10 | 0.924±0.053 | 0.0033±0.0018 |
|  | DL | Dali, Yunnan Province | 2012/6/30 | 100.3272° | 25.5280° | 2034.88 | Tobacco | 15 | 6 | 0.810±0.078 | 0.0025±0.0014 |
|  | XAW | Xuanwei, Yunnan Province | 2012/6/28 | 104.0684° | 26.1178° | 1994.69 | Tobacco | 14 | 10 | 0.923±0.060 | 0.0024±0.0014 |
|  | YA | Yong'an, Fujian Province | 2012/8/13 | 117.6182° | 25.8775° | 721.00 | Tobacco | 14 | 10 | 0.923±0.060 | 0.0032±0.0018 |
|  | HZ | Hezhou, Guangxi Province | 2012/8/17 | 111.2241° | 24.4830° | 181.00 | Tobacco | 13 | 9 | 0.936±0.051 | 0.0031±0.0018 |
|  | XIX | Xinxiang, Henan Province | 2012/8/22 | 113.9241° | 35.2748° | 83.00 | Tobacco | 14 | 11 | 0.956±0.045 | 0.0028±0.0016 |
|  | GY | Guiyang, Guizhou Province | 2012/8/24 | 106.8516° | 26.5005° | 1535.00 | Tobacco | 14 | 9 | 0.923±0.050 | 0.0025±0.0014 |
|  | ZY | Zunyi, Guizhou Province | 2012/8/25 | 106.8430° | 27.8404° | 979.00 | Tobacco | 14 | 11 | 0.934±0.061 | 0.0029±0.0016 |
|  | ZHY | Zhenyuan, Guizhou Province | 2012/8/26 | 108.3688° | 27.0466° | 692.00 | Tobacco | 15 | 8 | 0.848±0.071 | 0.0021±0.0012 |
|  | GM | Gaomi, Shandong Province | 2012/9/5 | 119.5456° | 36.2265° | 63.00 | Tobacco | 13 | 9 | 0.910±0.068 | 0.0023±0.0014 |
|  | TS | Tangshan, Hebei Province | 2012/9/6 | 118.2865° | 39.8935° | 87.00 | Tobacco | 9 | 5 | 0.861±0.087 | 0.0023±0.0014 |
|  | WEX | Wenxi, Shanxi Province | 2012/9/8 | 111.0742° | 35.3314° | 619.00 | Tobacco | 13 | 6 | 0.769±0.103 | 0.0017±0.0010 |
|  | RY | Ruyuan, Guangdong Province | 2013/6/5 | 114.1030° | 24.9977° | 107.00 | Tobacco | 27 | 13 | 0.841±0.059 | 0.0027±0.0015 |
|  | TX | Tongxiang, Zhejiang Province | 2013/7/28 | 120.4644° | 30.6646° | 0.36 | Tobacco | 13 | 5 | 0.705±0.122 | 0.0012±0.0008 |
|  | SZ | Shengzhou, Zhejiang Province | 2013/7/31 | 120.7011° | 29.5343° | 28.90 | Tobacco | 13 | 8 | 0.923±0.050 | 0.0034±0.0019 |
|  | JY | Jiangyin, Jiangsu Province | 2013/7/27 | 120.1594° | 31.8856° | 5.88 | Sesame | 15 | 10 | 0.943±0.040 | 0.0034±0.0019 |
|  | LX | Lanxi, Zhejiang Province | 2013/8/2 | 119.2727° | 29.1841° | 66.60 | Sesame | 11 | 8 | 0.891±0.092 | 0.0031±0.0018 |
|  | DZ | Dongzhi, Anhui Province | 2013/8/4 | 116.9691° | 30.3315° | 17.80 | Sesame | 15 | 11 | 0.952±0.040 | 0.0040±0.0022 |
|  | CHS | Changsha, Hunan Province | 2013/9/1 | 113.0517° | 28.2041° | 51.00 | Sesame | 11 | 6 | 0.800±0.114 | 0.0022±0.0013 |
|  | FC | Fengcheng, Jiangxi Province | 2013/9/3 | 115.7278° | 28.1647° | 32.00 | Sesame | 15 | 13 | 0.981±0.031 | 0.0046±0.0025 |
|  | BB | Bengbu, Anhui Province | 2013/8/7 | 117.4413° | 33.2981° | 26.30 | Tobacco | 15 | 9 | 0.905±0.054 | 0.0037±0.0021 |
|  | NG | Ningguo, Anhui Province | 2013/8/6 | 118.8785° | 30.7643° | 23.80 | Tobacco | 15 | 9 | 0.933±0.040 | 0.0033±0.0018 |
|  | XUC | Xuchang, Henan Province | 2013/8/10 | 113.6311° | 33.9623° | 80.60 | Tobacco | 15 | 10 | 0.924±0.053 | 0.0017±0.0010 |
|  | DAC | Dancheng, Henan Province | 2013/8/11 | 115.0455° | 33.6839° | 50.70 | Tobacco | 15 | 12 | 0.971±0.033 | 0.0038±0.0021 |
|  | DEZ | Dengzhou, Henan Province | 2013/8/14 | 111.9569° | 32.6502° | 141.00 | Tobacco | 15 | 8 | 0.867±0.067 | 0.0032±0.0018 |
|  | XY | Xiangyang, Hubei Province | 2013/8/16 | 112.6172° | 32.1760° | 122.00 | Tobacco | 14 | 9 | 0.934±0.045 | 0.0019±0.0011 |
|  | SL | Shangluo, Shaanxi Province | 2013/8/18 | 110.0602° | 34.0701° | 1020.00 | Tobacco | 14 | 8 | 0.769±0.120 | 0.0030±0.0017 |
|  | SYA | Yan'an, Shaanxi Province | 2013/8/22 | 109.5576° | 36.0937° | 1090.00 | Tobacco | 14 | 10 | 0.956±0.038 | 0.0036±0.0020 |
|  | XXA | Xi'an, Shaanxi Province | 2013/8/24 | 108.1847° | 34.1429° | 447.00 | Tobacco | 14 | 7 | 0.846±0.074 | 0.0013±0.0008 |
|  | GUY | Guangyuan, Sichuan Province | 2013/9/9 | 105.5590° | 32.1855° | 779.00 | Tobacco | 14 | 9 | 0.934±0.045 | 0.0031±0.0017 |
|  | HX | Huixian, Gansu Province | 2013/9/11 | 106.0272° | 33.7842° | 997.00 | Tobacco | 11 | 6 | 0.873±0.071 | 0.0024±0.0014 |
|  | LZ | Lanzhou, Gansu Province | 2013/9/12 | 104.1678° | 35.9163° | 1770.00 | Tobacco | 15 | 8 | 0.876±0.067 | 0.0033±0.0018 |
|  | LF | Langfang, Hebei Province | 2011/4/2 | 116.7634° | 39.4842° | 48.60 | Tobacco | 10 | 5 | 0.844±0.080 | 0.0013±0.0008 |

N, Sample size; HN, number of haplotype; Hd, haplotype diversity; Pi, nucleotide diversity.

**Supplementary Table S2.** **Pairwise *F_ST_* values for 37 populations of *Nesidiocoris tenuis* based on the combined mitochondrial dataset.**

|  | HZ | ZHY | GY | ZY | YUX | XAW | DL | GUY | HNA | NG | SZ | TX | YA | RY | XY | DEZ | DAC | XUC | XIX | BB | SL | XXA | SYA | HX | LZ | WEX | GM | LF | TS | JY | FC | DZ | LX | CHS | XICH | QJ | KM |
| --- | --- | --- | --- | --- | --- | --- | --- | --- | --- | --- | --- | --- | --- | --- | --- | --- | --- | --- | --- | --- | --- | --- | --- | --- | --- | --- | --- | --- | --- | --- | --- | --- | --- | --- | --- | --- | --- |
| HZ | - |  |  |  |  |  |  |  |  |  |  |  |  |  |  |  |  |  |  |  |  |  |  |  |  |  |  |  |  |  |  |  |  |  |  |  |  |
| ZHY | -0.035 | - |  |  |  |  |  |  |  |  |  |  |  |  |  |  |  |  |  |  |  |  |  |  |  |  |  |  |  |  |  |  |  |  |  |  |  |
| GY | -0.015 | 0.006 | - |  |  |  |  |  |  |  |  |  |  |  |  |  |  |  |  |  |  |  |  |  |  |  |  |  |  |  |  |  |  |  |  |  |  |
| ZY | -0.056 | -0.029 | -0.023 | - |  |  |  |  |  |  |  |  |  |  |  |  |  |  |  |  |  |  |  |  |  |  |  |  |  |  |  |  |  |  |  |  |  |
| YUX | -0.051 | 0.007 | -0.006 | -0.056 | - |  |  |  |  |  |  |  |  |  |  |  |  |  |  |  |  |  |  |  |  |  |  |  |  |  |  |  |  |  |  |  |  |
| XAW | -0.006 | -0.026 | -0.002 | -0.028 | 0.007 | - |  |  |  |  |  |  |  |  |  |  |  |  |  |  |  |  |  |  |  |  |  |  |  |  |  |  |  |  |  |  |  |
| DL | -0.064 | -0.036 | -0.007 | -0.059 | -0.049 | -0.020 | - |  |  |  |  |  |  |  |  |  |  |  |  |  |  |  |  |  |  |  |  |  |  |  |  |  |  |  |  |  |  |
| GUY | -0.054 | -0.025 | -0.044 | -0.064 | -0.054 | -0.027 | -0.052 | - |  |  |  |  |  |  |  |  |  |  |  |  |  |  |  |  |  |  |  |  |  |  |  |  |  |  |  |  |  |
| HNA | -0.037 | -0.015 | -0.035 | -0.056 | -0.044 | -0.028 | -0.038 | -0.068 | - |  |  |  |  |  |  |  |  |  |  |  |  |  |  |  |  |  |  |  |  |  |  |  |  |  |  |  |  |
| NG | -0.045 | -0.010 | -0.050 | -0.048 | -0.043 | -0.007 | -0.036 | -0.065 | -0.058 | - |  |  |  |  |  |  |  |  |  |  |  |  |  |  |  |  |  |  |  |  |  |  |  |  |  |  |  |
| SZ | -0.023 | 0.022 | 0.066 | -0.029 | -0.032 | 0.024 | -0.021 | -0.030 | -0.022 | 0.001 | - |  |  |  |  |  |  |  |  |  |  |  |  |  |  |  |  |  |  |  |  |  |  |  |  |  |  |
| TX | 0.056 | 0.023 | 0.032 | 0.015 | 0.060 | -0.021 | 0.038 | 0.009 | -0.004 | 0.020 | 0.083 | - |  |  |  |  |  |  |  |  |  |  |  |  |  |  |  |  |  |  |  |  |  |  |  |  |  |
| YA | -0.050 | -0.039 | 0.012 | -0.053 | -0.034 | -0.021 | -0.052 | -0.046 | -0.038 | -0.025 | -0.044 | 0.024 | - |  |  |  |  |  |  |  |  |  |  |  |  |  |  |  |  |  |  |  |  |  |  |  |  |
| RY | -0.033 | -0.014 | 0.034 | -0.032 | -0.019 | 0.006 | -0.037 | -0.028 | -0.017 | -0.008 | -0.026 | 0.034 | -0.041 | - |  |  |  |  |  |  |  |  |  |  |  |  |  |  |  |  |  |  |  |  |  |  |  |
| XY | 0.029 | 0.005 | -0.007 | 0.012 | 0.051 | -0.013 | 0.025 | -0.007 | -0.007 | 0.000 | 0.075 | -0.040 | 0.014 | 0.029 | - |  |  |  |  |  |  |  |  |  |  |  |  |  |  |  |  |  |  |  |  |  |  |
| DEZ | -0.048 | -0.005 | 0.022 | -0.053 | -0.054 | 0.004 | -0.047 | -0.050 | -0.041 | -0.028 | -0.061 | 0.059 | -0.052 | -0.036 | 0.052 | - |  |  |  |  |  |  |  |  |  |  |  |  |  |  |  |  |  |  |  |  |  |
| DAC | -0.040 | 0.011 | 0.016 | -0.040 | -0.047 | 0.021 | -0.033 | -0.047 | -0.037 | -0.030 | -0.056 | 0.068 | -0.040 | -0.025 | 0.048 | -0.059 | - |  |  |  |  |  |  |  |  |  |  |  |  |  |  |  |  |  |  |  |  |
| XUC | 0.055 | 0.011 | 0.054 | 0.028 | 0.075 | -0.012 | 0.043 | 0.019 | 0.011 | 0.036 | 0.069 | -0.035 | 0.015 | 0.033 | -0.026 | 0.057 | 0.064 | - |  |  |  |  |  |  |  |  |  |  |  |  |  |  |  |  |  |  |  |
| XIX | 0.015 | 0.040 | -0.052 | -0.001 | 0.014 | 0.010 | 0.026 | -0.033 | -0.034 | -0.040 | 0.068 | 0.028 | 0.029 | 0.055 | -0.007 | 0.037 | 0.024 | 0.048 | - |  |  |  |  |  |  |  |  |  |  |  |  |  |  |  |  |  |  |
| BB | -0.030 | 0.025 | -0.010 | -0.039 | -0.049 | 0.010 | -0.023 | -0.052 | -0.045 | -0.043 | -0.024 | 0.060 | -0.019 | 0.001 | 0.046 | -0.038 | -0.040 | 0.072 | -0.007 | - |  |  |  |  |  |  |  |  |  |  |  |  |  |  |  |  |  |
| SL | -0.053 | -0.005 | 0.013 | -0.059 | -0.062 | -0.004 | -0.054 | -0.050 | -0.041 | -0.029 | -0.049 | 0.061 | -0.048 | -0.031 | 0.054 | -0.061 | -0.050 | 0.066 | 0.031 | -0.045 | - |  |  |  |  |  |  |  |  |  |  |  |  |  |  |  |  |
| XXA | 0.041 | -0.019 | 0.034 | 0.017 | 0.069 | -0.033 | 0.030 | 0.014 | 0.006 | 0.031 | 0.084 | -0.020 | 0.009 | 0.038 | -0.015 | 0.057 | 0.070 | -0.027 | 0.040 | 0.071 | 0.059 | - |  |  |  |  |  |  |  |  |  |  |  |  |  |  |  |
| SYA | -0.058 | 0.000 | -0.011 | -0.048 | -0.052 | 0.020 | -0.041 | -0.053 | -0.034 | -0.041 | -0.027 | 0.094 | -0.033 | -0.009 | 0.065 | -0.047 | -0.047 | 0.093* | 0.013 | -0.044 | -0.050 | 0.077 | - |  |  |  |  |  |  |  |  |  |  |  |  |  |  |
| HX | -0.062 | -0.069 | -0.043 | -0.065 | -0.037 | -0.051 | -0.063 | -0.068 | -0.059 | -0.053 | -0.012 | 0.002 | -0.060 | -0.033 | -0.018 | -0.040 | -0.029 | -0.002 | -0.016 | -0.025 | -0.043 | -0.031 | -0.043 | - |  |  |  |  |  |  |  |  |  |  |  |  |  |
| LZ | -0.050 | 0.007 | -0.017 | -0.055 | -0.063 | 0.007 | -0.042 | -0.061 | -0.052 | -0.052 | -0.029 | 0.055 | -0.031 | -0.014 | 0.045 | -0.051 | -0.048 | 0.070 | 0.000 | -0.055 | -0.055 | 0.064 | -0.056 | -0.040 | - |  |  |  |  |  |  |  |  |  |  |  |  |
| WEX | -0.013 | -0.038 | 0.022 | -0.041 | -0.003 | -0.051 | -0.030 | -0.033 | -0.038 | -0.006 | -0.005 | -0.025 | -0.043 | -0.018 | -0.003 | -0.020 | 0.002 | -0.021 | 0.033 | 0.010 | -0.021 | -0.041 | 0.016 | -0.059 | -0.004 | - |  |  |  |  |  |  |  |  |  |  |  |
| GM | -0.011 | -0.011 | 0.060 | 0.005 | 0.026 | 0.035 | -0.003 | 0.012 | 0.024 | 0.024 | 0.030 | 0.095* | -0.013 | 0.011 | 0.069 | 0.012 | 0.022 | 0.076* | 0.088* | 0.053 | 0.016 | 0.059 | 0.019 | -0.018 | 0.031 | 0.018 | - |  |  |  |  |  |  |  |  |  |  |
| LF | 0.050 | 0.004 | 0.127* | 0.049 | 0.090 | 0.031 | 0.046 | 0.066 | 0.067 | 0.085 | 0.075 | 0.083* | 0.011 | 0.034 | 0.072 | 0.056 | 0.076 | 0.045 | 0.149* | 0.113* | 0.074 | 0.039 | 0.099 | 0.022 | 0.097 | 0.012 | 0.044 | - |  |  |  |  |  |  |  |  |  |
| TS | -0.029 | -0.025 | 0.079 | -0.009 | 0.011 | 0.021 | -0.024 | -0.007 | 0.007 | 0.013 | -0.033 | 0.081 | -0.055 | -0.046 | 0.040 | -0.026 | -0.021 | 0.034 | 0.094 | 0.028 | -0.008 | 0.069 | 0.009 | -0.023 | 0.020 | 0.004 | -0.027 | 0.016 | - |  |  |  |  |  |  |  |  |
| JY | -0.005 | 0.020 | 0.011 | -0.012 | -0.004 | 0.013 | 0.000 | -0.038 | -0.033 | -0.025 | -0.019 | 0.032 | -0.020 | -0.005 | 0.002 | -0.017 | -0.030 | 0.021 | -0.009 | -0.019 | -0.005 | 0.048 | -0.001 | -0.018 | -0.011 | 0.009 | 0.050 | 0.088* | -0.019 | - |  |  |  |  |  |  |  |
| FC | -0.036 | 0.030 | 0.001 | -0.031 | -0.047 | 0.035 | -0.020 | -0.044 | -0.032 | -0.038 | -0.031 | 0.077 | -0.017 | 0.000 | 0.056 | -0.041 | -0.050 | 0.084 | 0.005 | -0.047 | -0.038 | 0.088 | -0.050 | -0.017 | -0.050 | 0.028 | 0.038 | 0.105* | 0.008 | -0.023 | - |  |  |  |  |  |  |
| DZ | -0.045 | 0.033 | 0.041 | -0.021 | -0.037 | 0.063 | -0.018 | -0.018 | 0.004 | -0.008 | -0.020 | 0.136* | -0.014 | 0.007 | 0.108 | -0.033 | -0.035 | 0.133* | 0.065 | -0.021 | -0.037 | 0.124* | -0.056 | -0.004 | -0.037 | 0.054 | 0.035 | 0.115* | 0.013 | 0.026 | -0.038 | - |  |  |  |  |  |
| LX | 0.055 | 0.072 | 0.090* | 0.041 | 0.058 | 0.053 | 0.056 | 0.018 | 0.024 | 0.041 | 0.006 | 0.058 | 0.015 | 0.024 | 0.027 | 0.029 | 0.016 | 0.035 | 0.066 | 0.044 | 0.050 | 0.101* | 0.069 | 0.044 | 0.059 | 0.053 | 0.097* | 0.110* | -0.018 | -0.037 | 0.032 | 0.090 | - |  |  |  |  |
| CHS | -0.045 | -0.057 | 0.040 | -0.022 | 0.009 | -0.002 | -0.032 | -0.020 | -0.007 | -0.004 | -0.010 | 0.069 | -0.055 | -0.031 | 0.030 | -0.021 | -0.012 | 0.025 | 0.063 | 0.023 | -0.009 | 0.017 | -0.011 | -0.058 | 0.008 | -0.019 | -0.021 | 0.006 | -0.076 | -0.003 | 0.012 | 0.006 | 0.042 | - |  |  |  |
| XICH | 0.034 | 0.145* | 0.099* | 0.052 | 0.009 | 0.154* | 0.070 | 0.038 | 0.057 | 0.043 | 0.029 | 0.247* | 0.066 | 0.105* | 0.210* | 0.021 | -0.003 | 0.243* | 0.108* | 0.012 | 0.028 | 0.240* | -0.019 | 0.081 | 0.006 | 0.156* | 0.129* | 0.238* | 0.121 | 0.079 | -0.028 | -0.022 | 0.153* | 0.118* | - |  |  |
| QJ | 0.057 | 0.134* | 0.129* | 0.070 | 0.051 | 0.153* | 0.078 | 0.071 | 0.086 | 0.079 | 0.061 | 0.215* | 0.073 | 0.102* | 0.189* | 0.050 | 0.037 | 0.211* | 0.144* | 0.075 | 0.061 | 0.214* | 0.042 | 0.093 | 0.054 | 0.143* | 0.119* | 0.189* | 0.104 | 0.099* | 0.027 | 0.037 | 0.149* | 0.110* | 0.025 | - |  |
| KM | 0.071 | 0.170* | 0.153* | 0.091* | 0.053 | 0.181* | 0.100* | 0.089* | 0.107* | 0.097* | 0.061 | 0.254* | 0.098* | 0.129* | 0.232* | 0.058 | 0.043 | 0.254* | 0.168* | 0.060 | 0.064 | 0.254* | 0.037 | 0.120* | 0.055 | 0.176* | 0.153* | 0.230* | 0.128* | 0.122* | 0.022 | 0.020 | 0.175* | 0.138* | -0.045 | 0.034 | - |

*P<0.05; **P<0.01; ***P<0.001.

**Supplementary Table S3. Estimates of population size and numbers of effective immigrants per generation between geographic districts based on the combined mitochondrial dataset.**

| **District, *i*** | ***θ*** | ***Nem*** | | | | | |
| --- | --- | --- | --- | --- | --- | --- | --- |
|  |  | **SD *i*** | **CD *i*** | **ND *i*** | **SWD *i*** | **Total in** | **Total out** |
| **South** | 0.00096 | - | 0.85 | 0.72 | 1.61 | 3.18 | **199.90** |
| **Central** | 0.04566 | **106.37** | **-** | **77.03** | **106.99** | **290.39** | 76.88 |
| **North** | 0.04113 | **91.55** | **75.08** | **-** | **90.79** | **257.42** | 78.58 |
| **Southwest** | 0.00121 | 1.98 | 0.95 | 0.83 | - | 3.76 | **199.39** |

*θ*, the mutation-scaled population size; *Nem*, effective number of migrants per generation; Total in, effective number of migrants entering into each district per generation; Total out, effective number of migrants leaving out of each district per generation.

**Supplementary Table S4.** **The primers used in this study.**

| Gene | Primer name | Nucleotide sequence (5’-3’) | Reference |
| --- | --- | --- | --- |
| *CoI* | CoI-F | TTTAGTGGAATCACAACTTTAG | Present study |
|  | CoI-R | CATTTTTACCTCTCTCTTGACT | Present study |
| *Cytb* | Cytb-F | AAACTTACACGCCAATGGAGCT | Present study |
|  | Cytb-R | GGTCGTGCTCCAATTCAGGTTA | Present study |
| *ND2* | ND2-F | CTCATCATAAGAACGATAGC | Present study |
|  | ND2-R | CCCAATCATTAGAGGAAC | Present study |
| ITS2 | ITS2-F | CTAAGCGGTGGATCACTCGG | Marcilla, 2001 |
|  | ITS2-R | GCACTATCAAGCAACACGACTC | Marcilla, 2001 |

**Figure S1. Map of sampling localities of *Nesidiocoris tenuis*.** Map was generated with ArcGIS 10.0 (<http://www.esri.com/software/arcgis/arcgis-for-desktop>) and modified with Adobe Photoshop CS6 (http://www.adobe.com/products/photoshop).


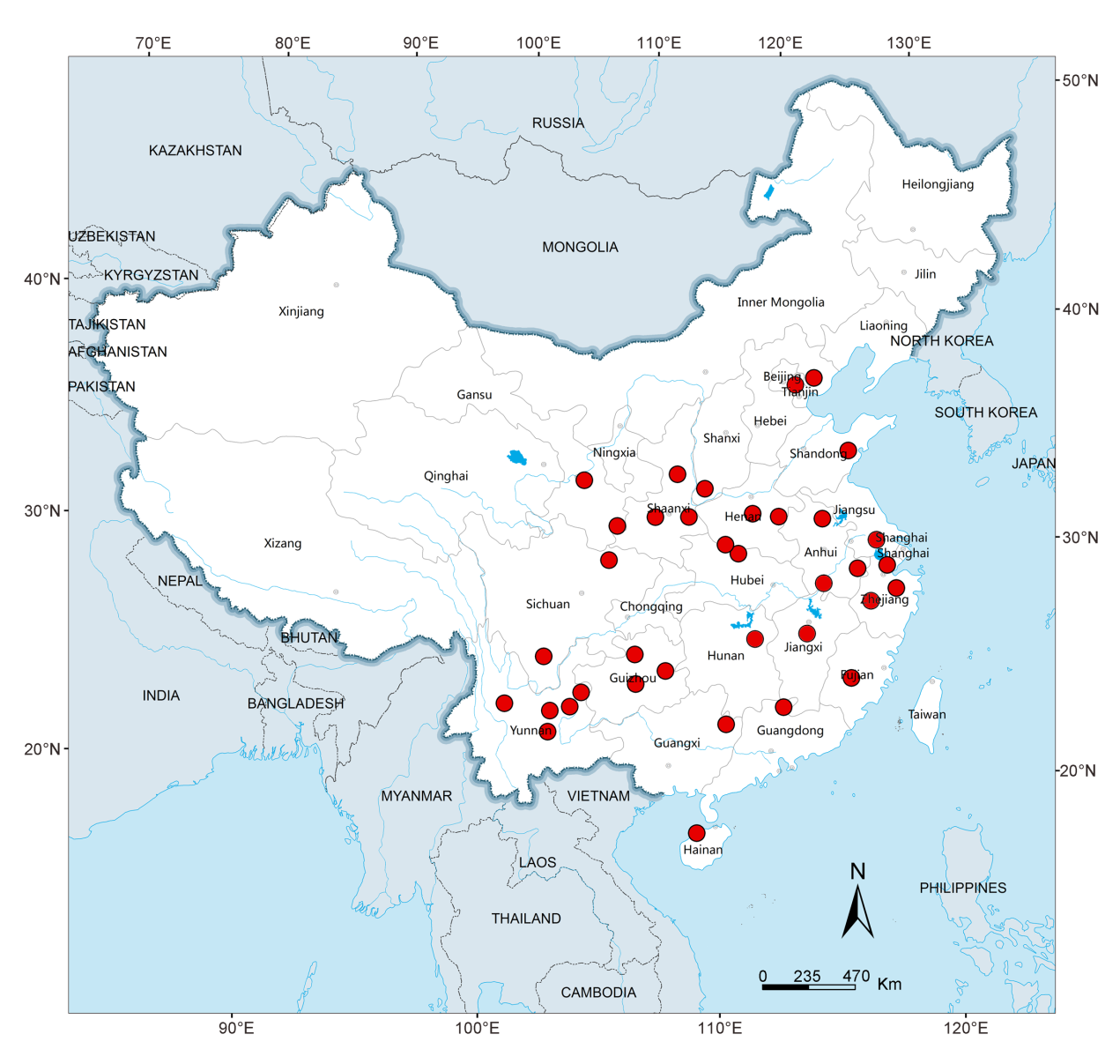


**Figure S2. Haplotype network for *Nesidiocoris tenuis* inferred from nuclear data.** The circle size of haplotype denotes the number of observed individuals.


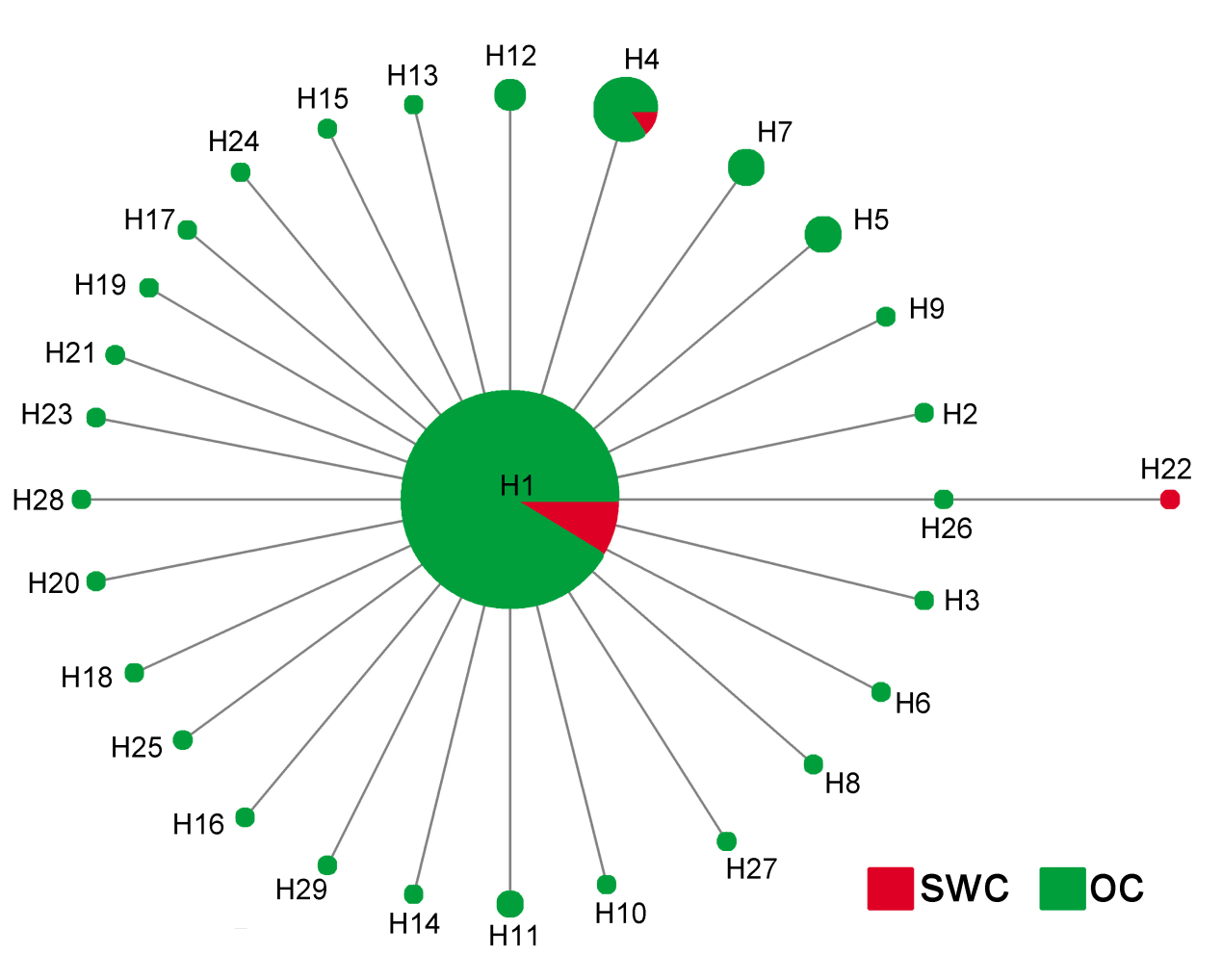


**Figure S3. Fixation indices by SAMOVA analysis based on nuclear data.**


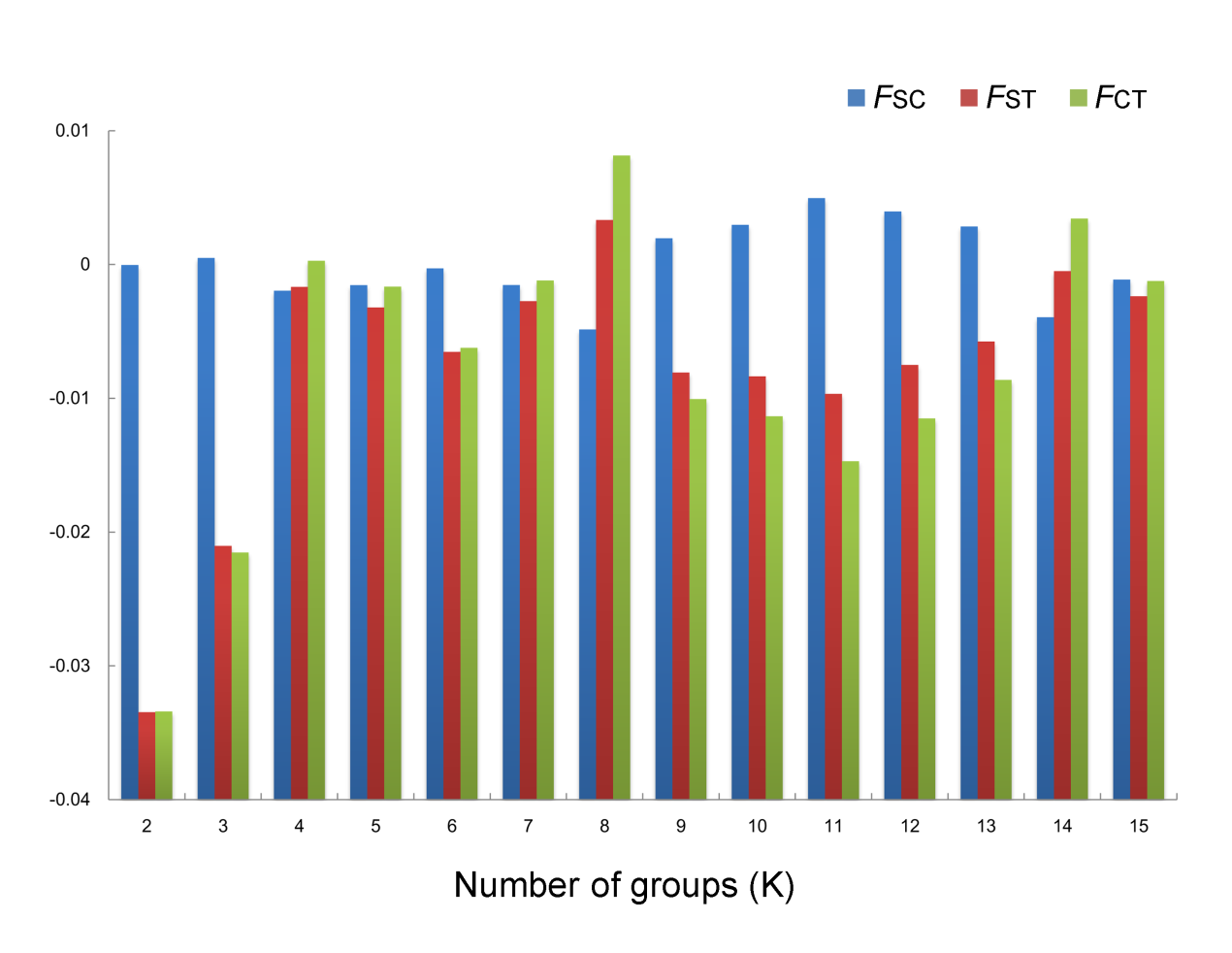


**Figure S4. Bayesian tree for the haplotypes of *Nesidiocoris tenuis* based on the combined mitochondrial dataset.** Blue color represents SWC group; yellow color represents OC group. When the haplotype was unique to one individual, the population name with a number denoted the haplotype name. When the haplotype was unique to same population, the “H” with the population name denoted the haplotype name. When the haplotype was belonged to different populations, the “H” with a number denoted the haplotype name.


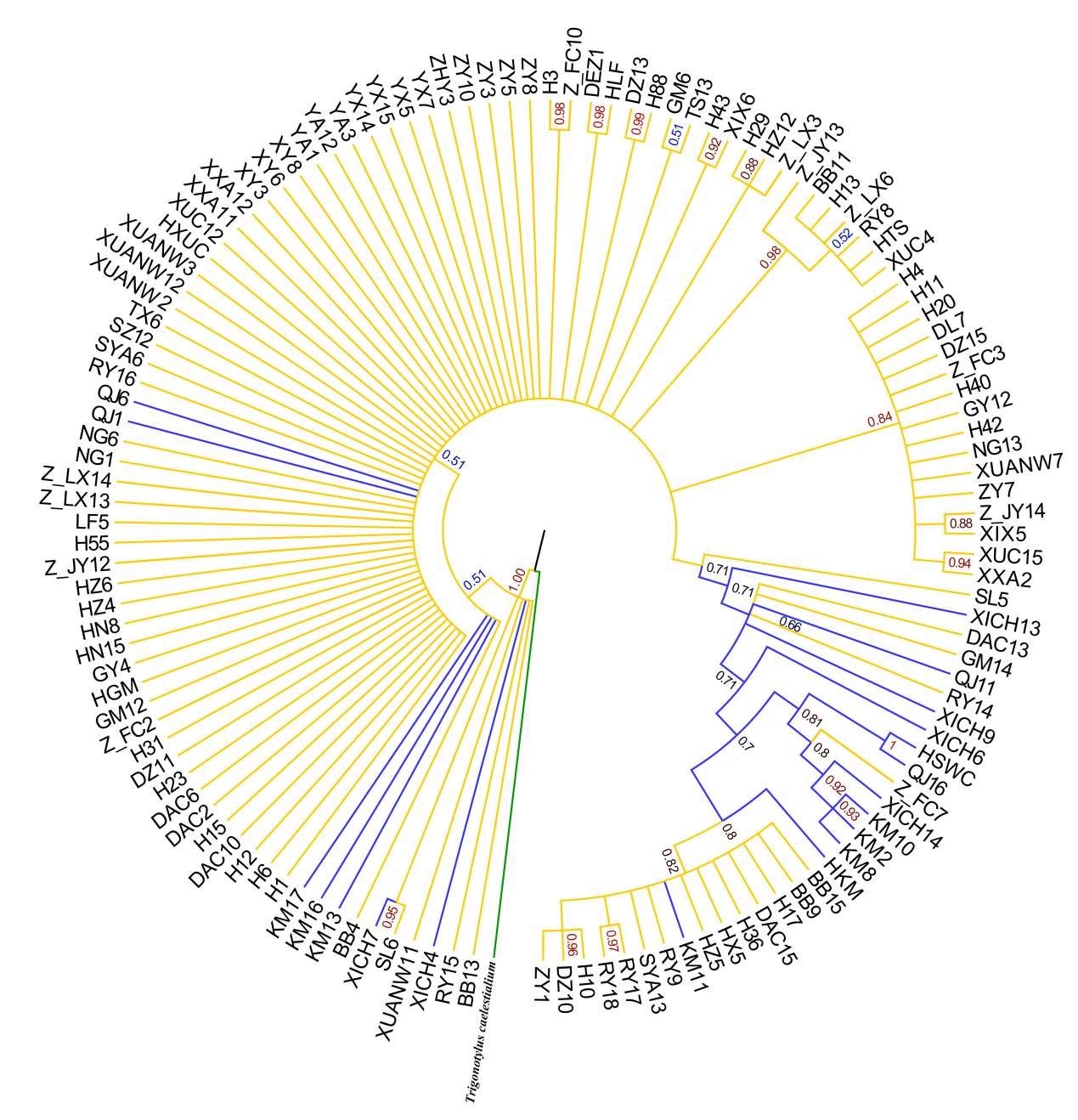

Supplement: Supplementary Information [file srep26755-s1.docx]
